# New Coleoptera records from New Brunswick, Canada: Stenotrachelidae, Oedemeridae, Meloidae, Myceteridae, Boridae, Pythidae, Pyrochroidae, Anthicidae, and Aderidae

**DOI:** 10.3897/zookeys.179.2629

**Published:** 2012-04-04

**Authors:** Reginald P. Webster, Jon D. Sweeney, Ian DeMerchant

**Affiliations:** 1 Natural Resources Canada, Canadian Forest Service - Atlantic Forestry Centre, 1350 Regent St., P.O. Box 4000, Fredericton, NB, Canada E3B 5P7

**Keywords:** Stenotrachelidae, Oedemeridae, Meloidae, Myceteridae, Boridae, Pythidae, Pyrochroidae, Anthicidae, Aderidae, new records, Canada, New Brunswick

## Abstract

We report 19 new species records for the faunal list of Coleoptera in New Brunswick, Canada, six of which are new records for the Maritime provinces, and one of which is new Canadian record. We also provide the first recent records for five additional species in New Brunswick. One new species of Stenotrachelidae, *Cephaloon ungulare* LeConte, is added to the New Brunswick faunal list. Additional records are provided for *Cephaloon lepturides* Newman, as well the first recent record of *Nematoplus collaris* LeConte. Two species of Oedemeridae, *Asclera puncticollis* (Say) and *Asclera ruficollis* (Say), are newly reported for New Brunswick, and additional locality and bionomic data are provided for *Calopus angustus* LeConte and *Ditylus caeruleus* (Randall). The records of *Ditylus caerulus* are the first recent records for the province. Three species of Meloidae, *Epicauta pestifera* Werner, *Lytta sayi* LeConte, and *Meloe augustcollis* Say are reported the first time for New Brunswick; *Epicauta pestifera* is newly recorded in Canada. *Lacconotus punctatus* LeConte and the family Mycteridaeis newly recorded for New Brunswick. The first recent records of *Borus unicolor* Say (Boridae) are reported from the province. One new species of Pythidae, *Pytho siedlitzi* Blair, and the first recent records of *Pytho niger* Kirby are added to the faunal list of New Brunswick. Three species of Pyrochroidae are newly reported for the province, including *Pedilus canaliculatus* (LeConte) and *Pedilus elegans* (Hentz), which are new for the Maritime provinces. Five species of Anthicidae and the first recent record of *Anthicus cervinus* LaFerté-Sénectére are newly reported for New Brunswick. *Anthicus melancholicus* LaFerté-Sénectère, *Sapintus pubescens* (LaFerté-Sénectère), *Notoxus bifasciatus* (LeConte), and *Stereopalpus rufipes* Casey are new to the Maritime provinces faunal list. *Ambyderus granularis* (LeConte) is removed from the faunal list of the province. Three species of Aderidae, *Vanonus huronicus* Casey, *Zonantes fasciatus* (Melsheimer), and *Zonantes pallidus* Werner, are newly recorded for New Brunswick; *Zonantes fasciatus* and *Vanonus huronicus* are new for the Maritime provinces’ faunal list. Collection data, bionomic data, and distribution maps are presented for all these species.

## Introduction

This paper treats new records from New Brunswick, Canada of a number of smaller families of beetles in the Tenebrionoidea: the Stenotrachelidae, Oedemeridae, Meloidae, Myceteridae, Boridae, Pythidae, Pyrochroidae, Anthicidae, and Aderidae. The fauna of most of these families from New Brunswick and Atlantic Canada was recently treated by Majka (2006) (Mycteridae, Boridae, Pythidae, Pyrochroidea), Majka (2011a) (Stenotrachelidae), Majka (2011b) (Anthicidae), Majka (2011c) (Aderidae), and Majka and Langor (2011) (Oedemeridae). Campbell (1991c) reported only three species of Meloidae from New Brunswick. However, there have been no recent treatments of this family from the region. Intensive sampling in New Brunswick by the first author since 2003 and records obtained from by-catch samples during a study to develop a general attractant for the detection of invasive species of Cerambycidae have yielded additional new provincial records in the above families. The purpose of this paper is to report on these new records. A brief synopsis of each family is included in the results below.

## Methods and conventions

The following records are based on specimens collected during a general survey by the first author to document the Coleoptera fauna of New Brunswick and from by-catch samples obtained during a study to develop a general attractant for the detection of invasive species of Cerambycidae. Additional records (including data from the Forest Insect and Disease Survey (FIDS) slips) were obtained from specimens contained in the collection belonging to Natural Resources Canada, Canadian Forest Service - Atlantic Forestry Centre, Fredericton, New Brunswick.

## Collection methods

Various methods were employed to collect the species reported in this study. Details are outlined in Webster et al. (2009, Appendix). Some specimens were collected from Lindgren funnel traps set in various forest types in New Brunswick between 2008 and 2011. These traps mimic tree trunks and are often effective for sampling species of Coleoptera that live in microhabitats associated with standing trees (Lindgren 1983). See Webster et al. (in press) for details of the methods used to deploy Lindgren 12-funnel traps and sample collection. A description of the habitat was recorded for all specimens collected during this survey. Locality and habitat data are presented exactly as on labels for each record. This information, as well as additional collecting notes, is summarized and discussed in collection and habitat data for each species.

### Distribution

Distribution maps, created using ArcMap and ArcGIS, are presented for each species in New Brunswick. Every species is cited with current distribution in Canada and Alaska, using abbreviations for the state, provinces, and territories. New records for New Brunswick are indicated in bold under Distribution in Canada and Alaska. Acronyms of collections examined or where specimens reside referred to in this study are as follows:

**Table:** 

**AK**	Alaska	**MB**	Manitoba
**YT**	Yukon Territory	**ON**	Ontario
**NT**	Northwest Territories	**QC**	Quebec
**NU**	Nunavut	**NB**	New Brunswick
**BC**	British Columbia	**PE**	Prince Edward Island
**AB**	Alberta	**NS**	Nova Scotia
**SK**	Saskatchewan	**NF & LB**	Newfoundland and Labrador*

* Newfoundland and Labrador are each treated separately under the current Distribution in Canada and Alaska.

The following abbreviations are used in the text:

AFCAtlantic Forestry Centre, Natural Resources Canada, Canadian Forest Service, Canada


CNCCanadian National Collection of Insects, Arachnids and Nematodes, Agriculture and Agri-Food Canada, Ottawa, Ontario, Canada


NBMNew Brunswick Museum, Saint John, New Brunswick, Canada


RWCReginald P. Webster Collection, Charters Settlement, New Brunswick, Canada


## Results

### Species accounts

All records below are species newly recorded for New Brunswick, Canada, unless noted otherwise (additional record). Species followed by ** are newly recorded from the Maritime provinces (New Brunswick, Nova Scotia, Prince Edward Island) of Canada; species followed by *** are newly recorded for Canada.

The classification of the Stenotrachelidae, Oedemeridae, Meloidae, Myceteridae, Boridae, Pythidae, Pyrochroidae, Anthicidae, and Aderidae follows Bouchard et al. (2011).

**Table 1.** Species of Stenotrachelidae, Oedemeridae, Meloidae, Myceteridae, Boridae, Pythidae, Pyrochroidae, Anthicidae, and Aderidae known from New Brunswick, Canada.

**Family Stenotrachelidae Thomson**
**Subfamily Cephaloinae LeConte**
*Cephaloon lepturides* Newman
*Cephaloon ungulare* LeConte*
**Subfamily Nematoplinae**
*Nematoplus collaris* LeConte
**Family Oedemeridae Latreille**
**Subfamily Calopodinae Costa**
*Calopus angustus* LeConte
**Subfamily Oedemerinae Latreille**
**Tribe Asclerini Gistel**
*Asclera puncticollis* (Say)*
*Asclera ruficollis* (Say)*
**Tribe Ditylini Mulsant**
*Ditylus caeruleus* (Randall)
**Tribe Nacerdini Mulsant**
*Nacerdes melanura* (Linnaeus)
**Family Meloidae Gyllenhal**
**Subfamily Meloinae Gyllenhal**
**Tribe Epicautini Parker and Böving**
*Epicauta murina* (LeConte)
*Epicauta pennsylvanica* (DeGeer)
*Epicauta pestifera* Werner***
**Tribe Lyttini Solier**
*Lytta sayi* LeConte**
**Tribe Meloini Gyllenhal**
*Meloe angusticollis* Say*
*Meloe impressus* Kirby
**Family Mycteridae Oken**
**Subfamily Eurypinae Thomso**
*Lacconotus punctatus* LeConte*
**Family Boridae Thomson**
*Borus unicolor* Say
*Lecontia discicollis* (LeConte)
**Family Pythidae Solier**
*Priognathus monilicornis* (Randall)
*Pytho americanus* Kirby
*Pytho niger* Kirby
*Pytho seidlitzi* Blair*
*Pytho strictus* LeConte
**Family Pyrochroidae**
**Subfamily Pedilinae Lacordaire**
*Pedilus canaliculatus* (LeConte)**
*Pedilus elegans* (Hentz)**
*Pedilus lugubris* (Say)
**Subfamily Pyrochroinae Latreille**
*Dendroides canadensis* Latreille
*Dendroides concolor* (Newman)
*Neopyrochroa femoralis* (LeConte)*
*Schizotus cervicalis* Newman
**Family Anthicidae Latreille**
**Subfamily Eurygeniinae LeConte**
*Stereopalpus rufipes* Casey**
**Subfamily Anthicinae Latreille**
*Amblyderus pallens* (LeConte
*Anthicus cervinus* LaFerté-Sénectère
*Anthicus coracinus* LeConte
*Anthicus flavicans* LeConte
*Anthicus haldemani* LeConte*
*Anthicus heroicus* Casey
*Anthicus melancholicus* LaFerté-Sénectère**
*Anthicus scabriceps* LeConte
*Malporus formicarius* (LaFerté-Sénectère)
*Omonadus floralis* (Linnaeus)
*Omonadus formicarius* (Goeze)
*Sapintus pubescens* (LaFerté-Sénectère)**
*Sapintus pusillus* (LaFerté-Sénectère)
**Subfamily Notoxinae Stephens**
*Notoxus anchora* Hentz
*Notoxus bifasciatus* (LeConte)**
**Family Aderidae Csiki**
**Tribe Euglenesini Seidlitz**
*Zonantes fasciatus* (Melsheimer)**
*Zonantes pallidus* Werner*
**Tribe Aderini Csiki**
*Vanonus wickhami* Casey
*Vanonus huronicus* Casey**

Notes: *New to province, **New to Maritime provinces, *** New to Canada.

### Family Stenotrachelidae Thomson, 1859

The Stenotrachelidae is a small family of beetles with only nine species known from Canada (Campbell 1991b). Little is known about the behavior of adults, other than that they are sometimes found on flowers and are most often captured in Malaise or flight-intercept traps (Young 2002a). Larvae develop in decaying wood, and some species such as *Nematophus* and possibly *Cephaloon* may be associated with logs infested with brown rot fungi (Young 2002a). *Nematoplus collaris* LeConte was the only species of Stenotrachelidae reported from New Brunswick by Campbell (1991b). Majka (2011c), in a review of this family for Atlantic Canada, added *Cephaloon lepturides* Newman. Here, we add another species, *Cephaloon ungulare* LeConte, to the New Brunswick fauna, as well as additional records for *Cephaloon lepturides* and the first recent record for *Nematoplus collaris* (Table 1).

### Subfamily Cephaloinae LeConte, 1862

#### Cephaloon lepturides

Newman, 1838

http://species-id.net/wiki/Cephaloon_lepturides

Map 1

##### Material examined.

**Additional New Brunswick records, Carleton Co.**, Meduxnekeag Valley Nature Preserve, 46.1957°N, 67.6803°W, 28.VI.2005, R. P. Webster, mixed forest, u.v. light trap (1, RWC); “Bell Forest”, 46.2200°N, 67.7231°W, 27.VI–5.VII.2008, R. P. Webster, Rich Appalachian hardwood forest with some conifers, Lindgren funnel trap (1, AFC). **Madawaska Co.**, Glasier Lake, 3.VII.1968 (D. Durling), 68–2-1721–02, on balsam fir (1, AFC). **Queens Co.**, Cranberry Lake P.N.A. (Protected Natural Area), 46.1125°N, 65.6075°W, 29.VI–7.VII.2011, M. Roy & V. Webster, old red oak forest, Lindgren funnel trap (1, NBM). **York Co.**, Fredericton, 29.VI.1936, R. E. Balch (1, AFC); Durham, 15.VII.1958, G. W. Barter (1, AFC), New Maryland (Charters Settlement), 45.8395°N, 66.7391°W, 23.VI.2003, 26.VI.2003, R. P. Webster, mixed forest, u.v. light (6, RWC); same locality data and collector, 19.VI.2004, mixed forest, on flowers of mountain ash (1, RWC); 15 km W of Tracy off Rt. 645, 45.6848°N, 66.8821°W, 8–15.VI.2009, 15–21.VI.2009, R. Webster & M.-A. Giguère, old red pine forest, Lindgren funnel traps (7, AFC); 14 km WSW of Tracy, S of Rt. 645, 45.6741°N, 66.8661°W, 22.V–2.VI.2010, R. Webster & C. MacKay, old mixed forest with red and white spruce, red and white pine, balsam fir, eastern white cedar, red maple, and *Populus* sp., Lindgren funnel trap (1, AFC).

##### Collection and habitat data.

This species was found in a rich Appalachian hardwood forest with some conifers, mixed forests, an old red oak (*Quercus rubra* L.) forest, and an old red pine (*Pinus resinosa* Ait.) forest. Specimens were collected from flowers of mountain ash (*Sorbus* sp.), at an ultraviolet light, on balsam fir (*Abies balsamea* (L.) Mill.), and in Lindgren funnel traps. In New Brunswick, adults were captured during May, June, and July.

##### Distribution in Canada and Alaska.

ON, QC, NB, NS, PE (Campbell 1991b; Majka 2011c). Majka (2011c) first reported this species from New Brunswick based on a specimen collected by E. Ouellete in Shediac, Westmorland Co. during July 1978. *Cephaloon lepturides* appears to be widespread in the province.

**Map 1.**
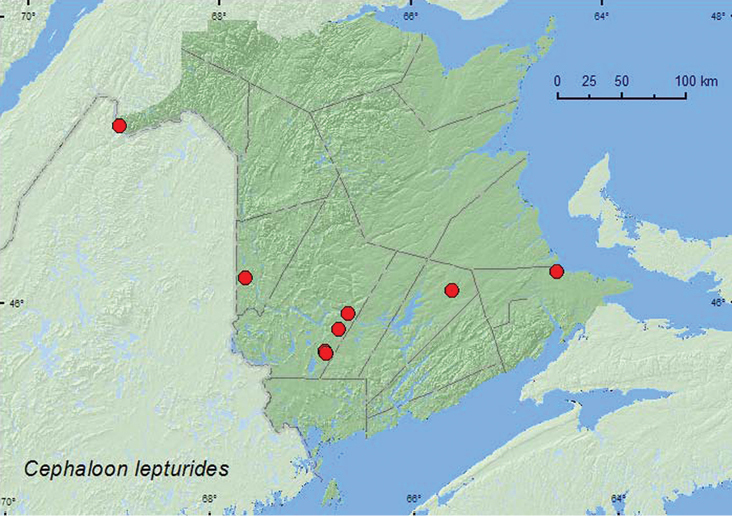
Collection localities in New Brunswick, Canada of *Cephaloon lepturides*.

#### Cephaloon ungulare

LeConte, 1874

http://species-id.net/wiki/Cephaloon_ungulare

Map 2

##### Material examined.

**New Brunswick, Restigouche, Co.**, Dionne Brook P.N.A., 47.9064°N, 68.3441°W, 27.VI–14.VII.2011, 14–28.VII.2011, M. Roy & V. Webster, old-growth balsam fir and white spruce forest, Lindgren funnel traps (3, RWC); same locality and collector but 47.9030°N, 68.3503°W, 14–28.VII.2011, old-growth northern hardwood forest, Lindgren funnel trap (1, NBM).

##### Collection and habitat data.

*Cephaloon ungulare* was collected in an old-growth balsam fir and white spruce (*Picea glauca* (Moench) Voss) forest and an old-growth northern hardwood forest. Adults were captured in Lindgren funnel traps during July. Most specimens of this species have been captured in flight-intercept or malaise traps in coniferous-dominated forests (Majka 2011c).

##### Distribution in Canada and Alaska.

ON, QC, **NB**, NS, PE, LB, NF (Campbell 1991b; Majka 2011c).

**Map 2.**
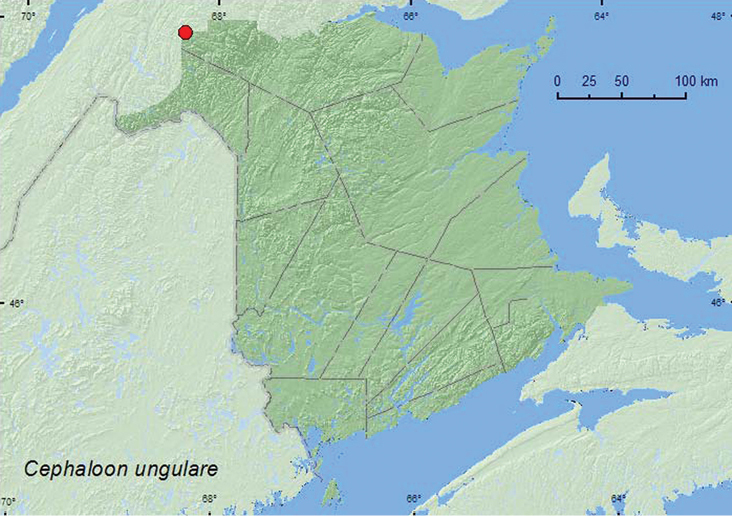
Collection localities in New Brunswick, Canada of *Cephaloon ungulare*.

### Subfamily Nematoplinae LeConte, 1862

#### Nematoplus collaris

LeConte, 1855

http://species-id.net/wiki/Nematoplus_collaris

Map 3

##### Material examined.

**Additional New Brunswick record, Restigouche, Co.**, Dionne Brook P.N.A., 47.9064°N, 68.3441°W, 27.VI–14.VII.2011, M. Roy & V. Webster, old-growth balsam fir and white spruce forest, flight intercept trap (1, RWC).

##### Collection and habitat data.

One individual of this species was captured between late June and mid July in a flight-intercept trap deployed in an old-growth balsam fir and white spruce forest.

##### Distribution in Canada and Alaska.

ON, QC, NB (Campbell 1991b). This species was previously known from New Brunswick on the basis of a specimen (in CNC) collected by J.N. Knull in Bathurst, Gloucester Co. during June 1913. The above record is the first recent record of this species from the province and from the Maritime provinces.

**Map 3.**
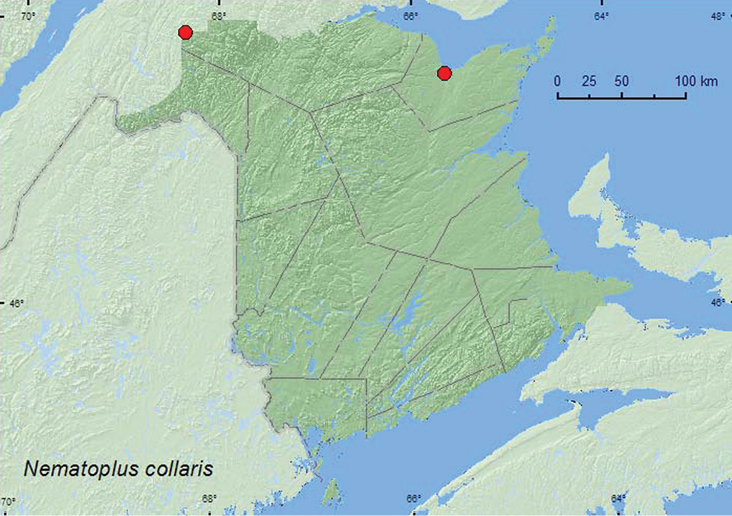
Collection localities in New Brunswick, Canada of *Nematoplus collaris*.

### Family Oedemeridae Latreille, 1810

The Oedemeridae (the false blister beetles) are usually found on flowers, foliage, and under driftwood and are often attracted to lights (Kriska 2002). Larvae typically occur in moist, decaying wood, including driftwood, in coastal species of oedemerids, and conifers for inland species (Kriska 2002). Campbell (1991e) reported only one species of Oedemeridae from New Brunswick; *Nacerdes melanura* (L). Majka and Langor (2011), in their review of the Oedermeridae of Atlantic Canada, added *Calopus angustus* LeConte and *Ditylus caeruleus* (Randall) to the faunal list of the province. Here, we report another two species, *Asclera puncticollis* (Say) and *Asclera ruficollis* (Say), and additional locality and habitat data for *Calopus angustus* and *Ditylus caeruleus* (Table 1).

### Subfamily Calopodinae Costa, 1852

#### Calopus angustus

LeConte, 1851

http://species-id.net/wiki/Calopus_angustus

Map 4

##### Material examined.

**Additional New Brunswick records, Carleton Co.**, Jackson Falls, Bell Forest, 46.2200°N, 67.7231°W, 6.V.2007, R. P. Webster, mature hardwood forest (with eastern white cedar), adult was in flight when collected (1, RWC); same locality and forest type, 23–28.IV.2009, 9–14.V.2009, R. P. Webster & M.-A. Giguère, Lindgren funnel traps (4, AFC, RWC). **Charlotte Co.**, 10 km NW of New River Beach, 45.2110°N, 66.6170°W, 30.IV–17.V.2010, R. Webster & V. Webster, old growth eastern white cedar forest, Lindgren funnel trap (1, AFC). **Northumberland Co.**, Priceville, 7.VI.1972, N. E. Carter, window trap (1, AFC). **Restigouche, Co.**, Dionne Brook P.N.A., 47.9030°N, 68.3503°W, 31.V–15.VI.2011, M. Roy & V. Webster, old-growth northern hardwood forest, Lindgren funnel traps (4, NBM, RWC); same locality and collectors but 47.9064°N, 68.3441°W, 31.V–15.VI.2011, old-growth white spruce and balsam fir forest, Lindgren funnel traps (15, AFC, NBM, RWC). **York Co.**, Fredericton, 20.IV.1966 (no collector given) (1, AFC); Charters Settlement, 45.8395°N, 66.7391°W, 1.V.1991, 4.V.1991, R. P. Webster, mixed forest (with eastern white cedar), u.v. light (2, NBM, RWC); 15 km W of Tracy off Rt. 645, 45.6848°N, 66.8821°W, 25.IV–4.V.2009, 11–19.V.2009, R. Webster & M.-A. Giguère, old red pine forest, Lindgren funnel traps (2, AFC, RWC); 14 km WSW of Tracy, S of Rt. 645, 45.6741°N, 66.8661°W, 26.IV–10.V.2010, R. Webster & C. MacKay, old mixed forest with red and white spruce, red and white pine, balsam fir, eastern white cedar, red maple, and *Populus* sp., Lindgren funnel trap (1, AFC).

##### Collection and habitat data.

Adults of *Calopus angustus* were collected in various forest types in New Brunswick, including hardwood forests with sugar maple (*Acer saccharum* Marsh.), American beech (*Fagus grandifolia* Ehrh.), eastern white cedar (*Thuja occidentalis* L.), an old-growth northern hardwood forest (white spruce, eastern white cedar, and balsam fir present), an old-growth eastern white cedar swamp, mixed forests, an old-growth white spruce and balsam fir forest, and an old red pine forest. Most adults were captured in Lindgren funnel traps. Some were also captured at an ultraviolet light. In western North America, Burke (1906) reared this species from a gallery of a living western cedar (*Thuja plicata* Don ex D. Don) and found larvae and pupae in dead and living branches of alpine fir (*Abies lasiocarpa* (Hook) Nutt.). This species probably uses related host trees, such as eastern white cedar and balsam fir, in our region. Adults were collected during April, May, and June, but most between late April and mid May.

##### Distribution in Canada and Alaska.

BC, AB, ON, QC, NB, NS (Campbell 1991e; Majka and Langor 2011). Majka and Langor (2011) reported this species for the first time for New Brunswick from one locality in Madawaska Co (East Iroquois River) and two localities in York Co. (Fredericton and Charters Settlement). This species is widespread and locally common in the province.

**Map 4.**
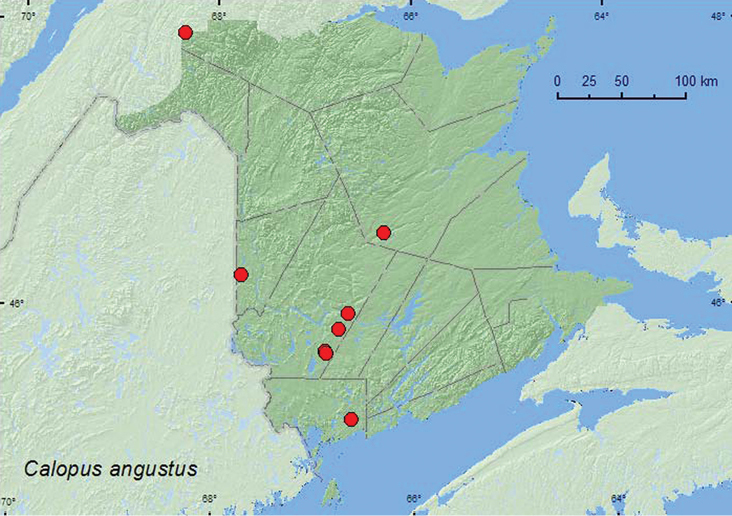
Collection localities in New Brunswick, Canada of *Calopus angustus*.

### Subfamily Oedemerinae Latreille, 1810

Tribe Asclerini Gistel, 1848

#### Asclera puncticollis

(Say, 1823)

http://species-id.net/wiki/Asclera_puncticollis

Map 5

##### Material examined.

**New Brunswick, Carleton Co.**, Jackson Falls, Bell Forest, 46.2200°N, 67.7231°W, 12–19.VI.2008, R. P. Webster, mature hardwood forest, Lindgren funnel trap (1, RWC); same locality and forest type but 23–28.IV.2009, 20–26.V.2009, R. Webster & M.-A. Giguère, Lindgren funnel traps (2, AFC); Meduxnekeag Valley Nature Preserve, 46.1890°N, 67.6766°W, 8.VI.2005, R. Webster & M.-A. Giguère, floodplain forest, on flowers of *Prunus virginiana* (1, RWC). **Queens Co.**, Cranberry Lake P.N.A., 46.1125°N, 65.6075°W, 12–21.V.2009, 21–27.V.2009, 5–11.VI.2009, R. Webster & M.-A. Giguère, mature red oak forest, Lindgren funnel traps (4, AFC, RWC); same locality data and forest type, 13–25.V.2011, 25.V–7.VI.2011, M. Roy & V. Webster, Lindgren funnel traps in forest canopy (8, AFC, NBM); Grand Lake Meadows P.N.A., 45.8227°N, 66.1209°W, 27.VI–5.VII.2011, M. Roy & V. Webster, old silver maple forest and seasonally flooded marsh, Lindgren funnel trap (1, NBM). **Sunbury Co.**, Acadia Research Forest, 45.9866°N, 66.3841°W, 25.V.–2.VI.2009, R. Webster & M.-A. Giguère, mature (110-year-old) red spruce forest with scattered red maple and balsam fir, Lindgren funnel trap (1, RWC). **York Co.**, Charters Settlement, 45.8395°N, 66.7391°W, 19.VI.2004, R. P. Webster, mixed forest, on lilac flowers (3, RWC); 15 km W of Tracy off Rt. 645, 45.6848°N, 66.8821°W, R. Webster & M.-A. Giguère, 25.V–1.VI.2009, 15–21.VI.2009, old red pine forest, Lindgren funnel traps (2, AFC); same locality and forest type but 18.V–4.VI.2010, 4–16.VI.2010, R. Webster & C. MacKay, Lindgren funnel traps (7, AFC, RWC); 14 km WSW of Tracy, S of Rt. 645, 45.6741°N, 66.8661°W, 16–30.VI.2010, R. Webster & C. MacKay, old mixed forest with red and white spruce, red and white pine, balsam fir, eastern white cedar, red maple, and *Populus* sp., Lindgren funnel trap (1, AFC).

##### Collection and habitat data.

*Asclera puncticollis*was found in a hardwood forest with sugar maple and American beech, a floodplain forest, an old red oak forest, an old silver maple (*Acer saccharinum* L.) swamp, an old mixed forest, an old red pine forest, and a mature red spruce forest. Adults were collected from choke cherry (*Prunus virginiana* L.) and lilac (*Syringa vulgaris* L.) flowers but most individuals were captured in Lindgren funnel traps. Adults were collected during April, May, June, and July.

##### Distribution in Canada and Alaska.

MB, ON, QC, **NB**, NS (Campbell 1991e; Majka and Langor 2011).

**Map 5.**
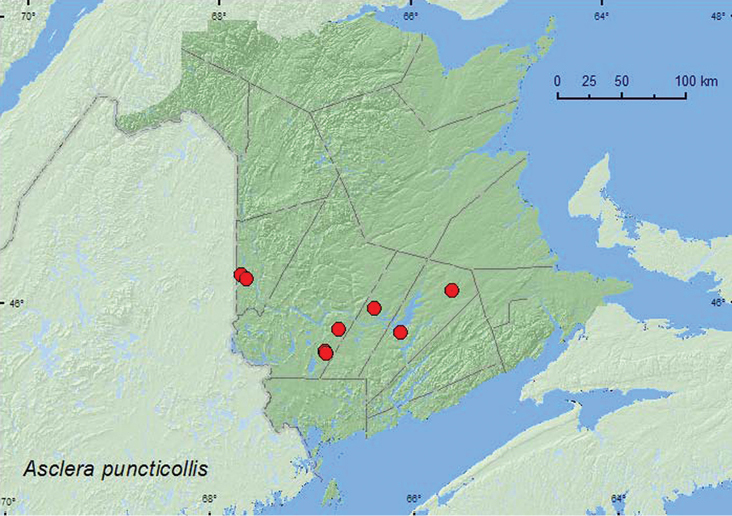
Collection localities in New Brunswick, Canada of *Asclera puncticollis*.

#### Asclera ruficollis

(Say, 1823)

http://species-id.net/wiki/Asclera_ruficollis

Map 6

##### Material examined.

**New Brunswick, Carleton Co.**, Jackson Falls, Bell Forest, 46.2252°N, 67.7190°W, 12.VII.2004, , K. Bredin, J. Edsall, & R. Webster, floodplain forest, sweeping foliage (1, RWC); same locality and habitat, 11.V.2005, R. P. Webster, on trout lily flower (2, NBM, RWC); same locality and collector but 46.2200°N, 67.7231°W, 19.IV.2005, mature hardwood forest, in leaf litter at base of tree (1, RWC); same locality and habitat, 20.VI.2005, R. Webster & M.-A. Giguère, on flowers of *Cornus* sp. (2, RWC); same locality, habitat, and collectors, 28.IV–9.V.2009, 20–26.V.2009, 1–8.VI.2009, 21–28.VI.2009, Lindgren funnel traps (6, AFC, RWC); Meduxnekeag Valley Nature Preserve, 46.1890°N, 67.6766°W, 8.VI.2005, R. Webster & M.-A. Giguère, floodplain forest, on flowers of *Prunus virginiana* (1, RWC). **York Co.**, Charters Settlement, 45.8395°N, 66.7391°W, 19.VI.2004, R. P. Webster, mixed forest, on lilac flowers (1, RWC); Canterbury, near Browns Mountain Fen, 45.8951°N, 67.6333°W, 10.VI.2005, R. Webster & M.-A. Giguère, mixed forest, on flowers of *Prunus virginiana* (1, RWC); Rt. 645 at Beaver Brook, 45.6830°N, 66.8679°W, 8.VII.2008, R. P. Webster, red maple and alder swamp, on flowers of *Ilex verticiliata* (winter berry) (1, RWC).

##### Collection and habitat data.

This species was found in a hardwood forest with sugar maple and American beech, a floodplain forest, and a mixed forest. Adults were collected from flowers of trout lily (*Erythronium americanum* Ker-Gawl.), lilac, *Cornus* sp., choke cherry, and winter berry (*Ilex verticiliata* (L.)). A few individuals were swept from foliage or sifted from leaf litter at the base of a tree; others were captured in Lindgren funnel traps. Majka and Langor (2011) reported this species from various flower species in Nova Scotia. Adults were captured during April, May, June, and July in New Brunswick.

##### Distribution in Canada and Alaska.

ON, QC, **NB**, NS (Campbell 1991e; Majka and Langor 2011).

**Map 6.**
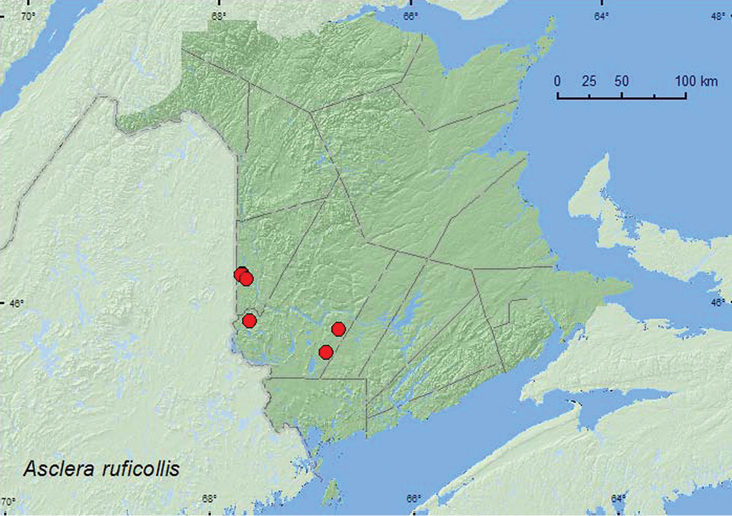
Collection localities in New Brunswick, Canada of *Asclera ruficollis*.

### Tribe Ditylini Mulsant, 1858

#### Ditylus caeruleus

(Randall, 1838)

http://species-id.net/wiki/Ditylus_caeruleus

Map 7

##### Material examined.

**Additional New Brunswick records, Carleton Co.**, 8 km SE of Benton, 14.VI.1990, R. P. Webster (1, NBM). **Restigouche Co.**, 12.1 km NNE of Kedgwick at Bologna Gulch, 47.77°N, 67.31°W, 13.VI.2000, R. P. Webster, sedge marsh (1, NBM); Stillwater Rd. at Stillwater Brook, 47.7320°N, 67.3376°W, 12.VI.2006, R. P. Webster, black spruce forest, in litter and moss near brook (1, RWC); NE jct. Little Tobique River and Red Brook, 47.4458°N, 67.0617°W**,** 13.VI.2006, R. P. Webster, alder swamp with eastern white cedar, in moss and grass litter near brook (1, RWC); 7.5 km S of Saint Arthur, 47.8283°N, 66.7654°W, 14.VI.2006, R. P. Webster (1, NBM); Jacquet River Gorge P.N.A., 47.7749°N, 66.1262°W, 23.VI.2008, R. P. Webster, mixed forest, adult in flight when collected (1, RWC); same locality but 47.8221°N, 66.0082°W, 13.V.2010, R. P. Webster, margin of *Carex* marsh, in leaf and grass litter under shrubs (1, NBM). **York Co.**, Charters Settlement, 45.8395°N, 66.7391°W, 13.VI.1993, R. P. Webster, mixed forest (1, RWC); Charters Settlement, 45.8331°N, 66.7279°W, 10.V.2010, R. P. Webster, beaver dam, among sticks, debris, and mud on dam (over 10 individuals observed) (2, RWC); Canterbury, near Browns Mountain Fen, 45.8951°N, 67.6333°W, 10.VI.2005, R. Webster & M.-A. Giguère, mixed forest, sweeping foliage on forest trail (1, RWC); 15 km W of Tracy off Rt. 645, 45.6837°N, 66.8809°W, 10.VI.2009, R. P. Webster, clear-cut (red pine), on red pine stump (1, RWC).

##### Collection and habitat data.

The larvae of *Ditylus* have been found in old wet cedar logs and the larval stage may last 3 years (Arnett 1951; Kriska 2002). In New Brunswick, this species was collected in a black spruce (*Picea mariana* (Mill.) B.S.P.) forest, a red pine forest, an alder (*Alnus* sp.) swamp, mixed forests, *Carex* marshes, and a beaver (*Castor canadensis* Kuhl.) dam. Adults were collected from leaf and grass litter and moss, by sweeping foliage, in flight, and on a red pine stump. Adults were common among sticks, debris, and mud within a beaver dam. Adults were collected during May and mid June.

##### Distribution in Canada and Alaska.

MB, ON, QC, NB, NS, NF (Campbell 1991e; Majka and Langor 2011). Majka and Langor (2011) first reported this species from New Brunswick based on specimens (in NBM) collected by W. McIntosh in Saint John during 1901. The above records are the first recent records of this species from the province. This species appears to be relatively common and widespread in New Brunswick.

**Map 7.**
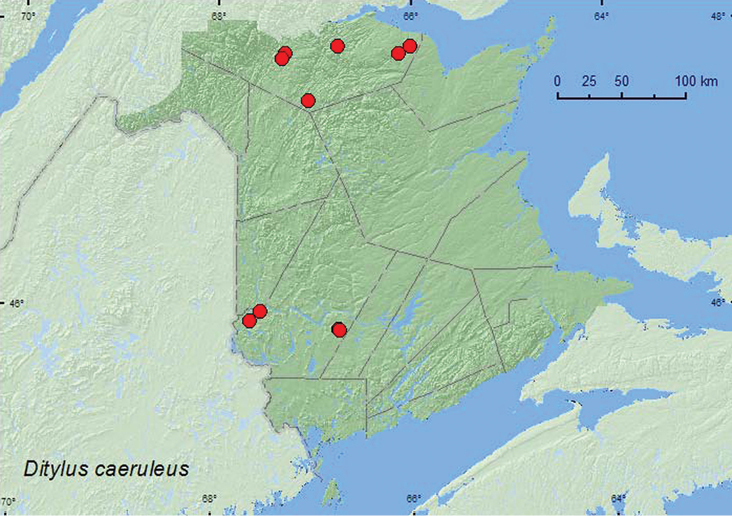
Collection localities in New Brunswick, Canada of *Ditylus caeruleus*.

### Family Meloidae Gyllenhal, 1810

Most adult Meloidae (the blister beetles) are phytophagous, found particularly on species of Asteraceae, Leguminosae, and Solanaceae (Pinto and Bologna 2002). The larvae are parasitoids on the provisions and immature stages of wild bees and eggs of grasshoppers. Campbell (1991c) reported 49 species and subspecies of Meloidae from Canada, most from the semiarid regions of the Prairie provinces and British Columbia. Only three species (*Meloe impressus* (Kirby), *Epicauta murina* (LeConte), and *Epicauta pennsylvanica* (DeGeer)), were reported from New Brunswick (Campbell 1991c). Here, we report *Epicauta pestifera* Werner, *Lytta sayi* LeConte, and *Meloe angusticollis* Say for the first time for New Brunswick (Table 1). *Epicauta pestifera* is newly recorded in Canada.

### Subfamily Meloinae Gyllenhal, 1810

Tribe Epicautini Parker and Böving, 1924

#### Epicauta pestifera

Werner, 1949***

http://species-id.net/wiki/Epicauta_pestifera

Map 8

##### Material examined.

**New Brunswick, Sunbury Co.**, 9.5 km NE jct. Rt. 101 & 645, 45.7586°N, 66.6755°W, 30.VIII.2008, R. P. Webster, old field with open sandy areas, sweeping cow vetch (1, RWC).

##### Collection and habitat data.

One individual was collected from cow vetch (*Vicia cracca* L.) in an old field with open sandy areas during late August.

##### Distribution in Canada and Alaska.

**ON**, **NB** (new Canadian records)**.** This species was not recorded from Canada by Campbell (1991c). There is one specimen in the CNC from Ontario from Elgin Co., Sparta, East Bridge Trail, 5 September 1992, Neva Carmichael.

**Map 8.**
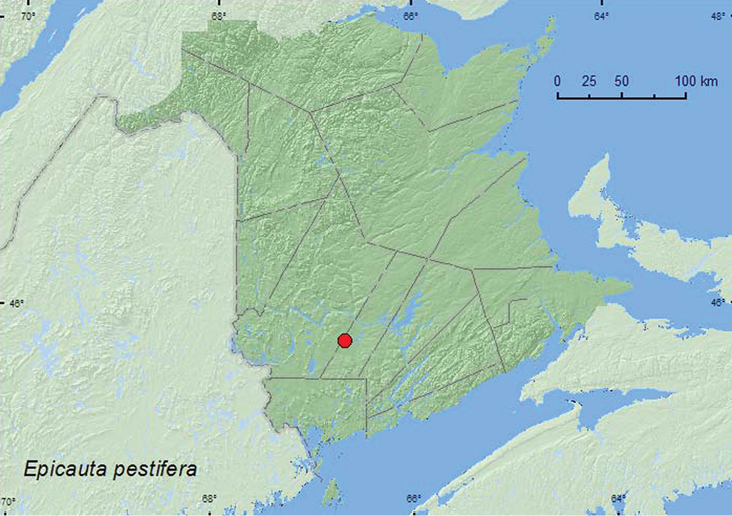
Collection localities in New Brunswick, Canada of *Epicauta pestifera*.

### Tribe Lyttini Solier, 1851

#### Lytta sayi

LeConte, 1853**

http://species-id.net/wiki/Lytta_sayi

Map 9

##### Material examined.

**New Brunswick, Gloucester Co.**, Bathurst, Daly Point Reserve, 16.VI.1996, R.P. Webster (1, RWC). **York Co.**, Durham, 27.V.1957, G. W. Barter, on willow (1, AFC); Harvey Station, 29.VI.1952, L. J. Simpson, choke cherry (2, AFC); Canterbury, 25.VI.1962, (Leon Thornton), black locust, 62–0697–01 (4, AFC); Longs Creek, 28.V.1963 (C. M. D.), on black cherry, 63–0111–01 (3, AFC); Charters Settlement, 45.8395°N, 66.7391°W, 19.VI.2004, R. P. Webster, mixed forest, on flowers of mountain ash (6, RWC); Upper Brockway, 45.5684°N, 67.0993°W, 3.VI.2005, R. P. Webster, (1, RWC).

##### Collection and habitat data.

Most adults of this species were collected from flowers in New Brunswick. These included black locust (*Robinia pseudoacacia* L.), choke cherry, and mountain ash. This species was collected during May and June.

##### Distribution in Canada and Alaska.

ON, QC, **NB** (Campbell 1991c)

**Map 9.**
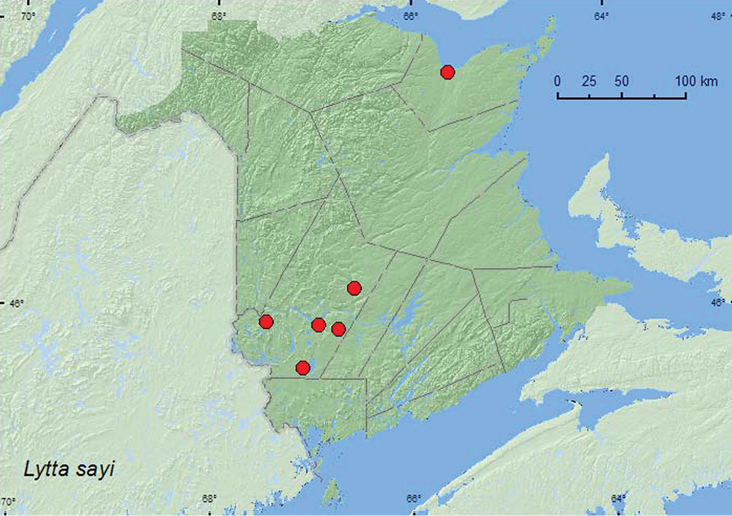
Collection localities in New Brunswick, Canada of *Lytta sayi*.

### Tribe Meloini Gyllenhal, 1810

#### Meloe angusticollis

Say, 1824

http://species-id.net/wiki/Meloe_angusticollis

Map 10

##### Material examined.

**New Brunswick, York Co.**,5.3 km SW of jct. Hwy 101 & Charters Settlement Rd., 4.V.1998, R. P. Webster (1, RWC).

##### Collection and habitat data.

No habitat data were included with the specimen. The adult was collected in early May.

##### Distribution in Canada and Alaska.

BC, AB, SK, MB, ON, QC, **NB**, NS (Campbell 1991c).

**Map 10.**
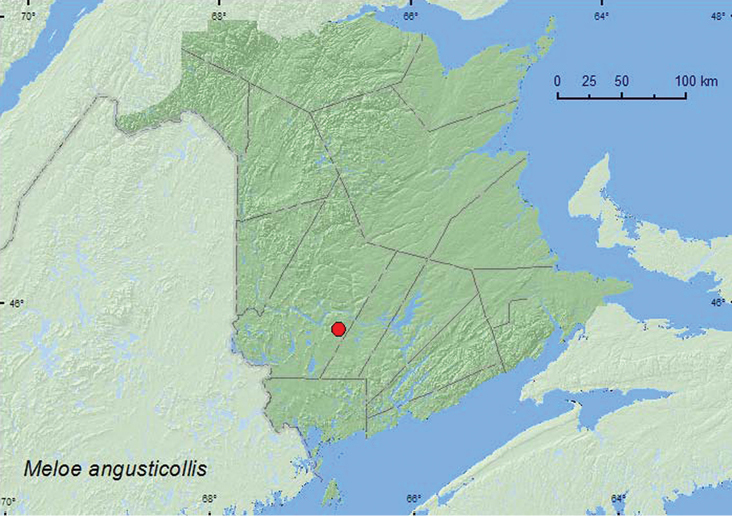
Collection localities in New Brunswick, Canada of *Meloe angusticollis*.

### Family Mycteridae Oken, 1843

The Mycteridae (the palm and flower beetles) of North America was reviewed by Pollock (2002a). Little is known about the natural history of members of this family occurring in Canada. *Mycterus* adults are often collected from flowers (Pollock 2002a). A western species of *Lacconotus* was collected from under the bark of dead poplar (*Populus* spp.) (Lawrence 1991), and it is likely that most species of Eurypinae (formerly Lacconotinae) live under bark of dead trees (Pollock 2002a). The habits of adults are little known. Only four species of this family are known from Canada (Campbell 1991d). *Lacconotus punctatus* LeConte and the family Mycteridae were newly reported for the Maritime provinces by Majka and Selig (2006). Here, we report this species and family for the first time for New Brunswick (Table 1).

### Subfamily Eurypinae Thomson, 1860

#### Lacconotus punctatus

LeConte, 1862

http://species-id.net/wiki/Lacconotus_punctatus

Map 11

##### Material examined.

**New Brunswick, Queens Co.**, Grand Lake Meadows P.N.A., 45.8227°N, 66.1209°W, 19–31.V.2010, R. Webster & C. MacKay, old silver maple forest with green ash and seasonally flooded marsh, Lindgren funnel trap (1, RWC); Cranberry Lake P.N.A., 46.1125°N, 65.6075°W, 25.V–7.VI.2011, 7–22.VI.2011, M. Roy & V. Webster, mature red oak forest, Lindgren funnel traps (2, RWC). **Sunbury Co.**, Acadia Research Forest, 45.9866°N, 66.3841°W, 2–9.VI.2009, R. Webster & M.-A. Giguère, mature (110-year-old) red spruce forest with scattered red maple and balsam fir, Lindgren funnel trap (1, AFC).

##### Collection and habitat data.

Specimens of *Lacconotus punctatus* from New Brunswick were captured in Lindgren funnel traps deployed in an old silver maple forest, an old red oak forest, and a 110-year-old red spruce forest. Adults were captured during May and June. Larvae of *Lacconotus* occur under bark of conifers and deciduous trees (Lawrence 1991).

##### Distribution in Canada and Alaska.

ON, QC, **NB**, NS (Campbell 1991c; Majka and Selig 2006).

**Map 11.**
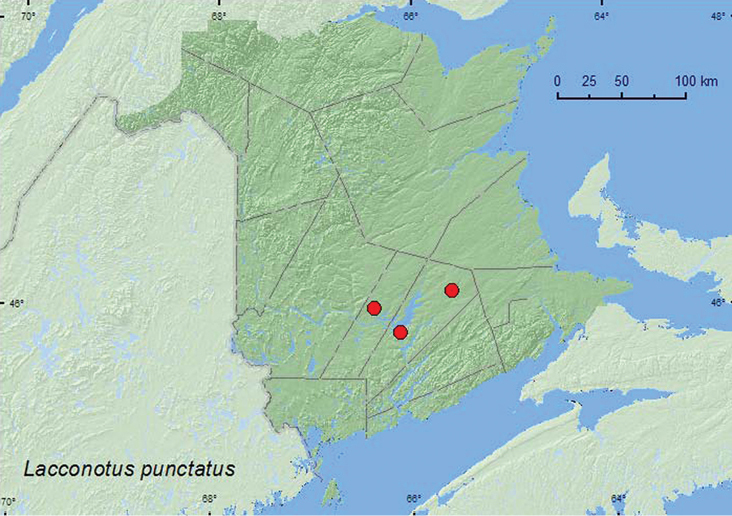
Collection localities in New Brunswick, Canada of *Lacconotus punctatus*.

### Family Boridae Thomson, 1859

The Boridae (the conifer bark beetles) is a small family of beetles represented by two species (*Borus unicolor* Say and *Lecontia discicollis* (LeConte)) in Canada and North America (Campbell 1991a; Pollock 2002b). The North American representatives of this family were reviewed by Pollock (2002b). Larvae of *Borus unicolor* inhabit in the subcortical region of dead, often standing or leaning, pines (*Pinus* sp.) and other coniferous species (Young 1991a). Larvae of *Lecontia discicollis* live in the subcortical region of fire-killed conifers (Young et al. 1996). Both species were reported by Majka (2006) for New Brunswick. *Borus unicolor* was reported for the first time for the province based on a specimen (in NBM) collected by W. McIntosh on 19 July 1901 in Saint John (Saint John Co.) (Majka 2006). Here, we report the first recent records of this uncommon species from the province (Table 1).

### Subfamily Borinae Thomson, 1859

#### Boros unicolor

Say 1827

http://species-id.net/wiki/Boros_unicolor

Map 12

##### Material examined.

**Additional New Brunswick records. Northumberland Co.**, Near the mouth of the (Big) Sevogle River (north of Big Hole), 18.VI.1941, H. Estey, from jack pine, beating (1, AFC). **York Co.**, 15 km W of Tracy off Rt. 645, 45.6848°N, 66.8821°W, 25.IV–4.V.2009, 19–25.V.2009, 8–15.VI.2009, 14–20.VII.2009, R. Webster & M.-A. Giguère, old red pine forest, Lindgren funnel traps (4, AFC, RWC); same locality and habitat data but 26.IV–10.V.2010, 10–26.V.2010, 18.V–2.VI.2010, 2–18.VI.2010, 18.V–2.VI.2010, 2–16.VI.2010, 30.VI–13.VII.2010, 13–27.VII.2010, 10–30.VIII.2010, R. Webster, C. MacKay, C. Hughes, & K. Burgess, Lindgren funnel traps (10, AFC, RWC).

##### Collection and habitat data.

Twenty-five specimens of this species are reported from New Brunswick. Most were captured in Lindgren funnel traps deployed in an old red pine forest. One individual was beaten from foliage of jack pine (*Pinus banksiana* Lamb.). Adults were captured during late April, May, June, July, and August.

##### Distribution in Canada and Alaska.

AB, SK, MB, ON, QC, NB (Campbell 1991e; Majka 2006). The records above are the first modern records of this species for the province.

**Map 12.**
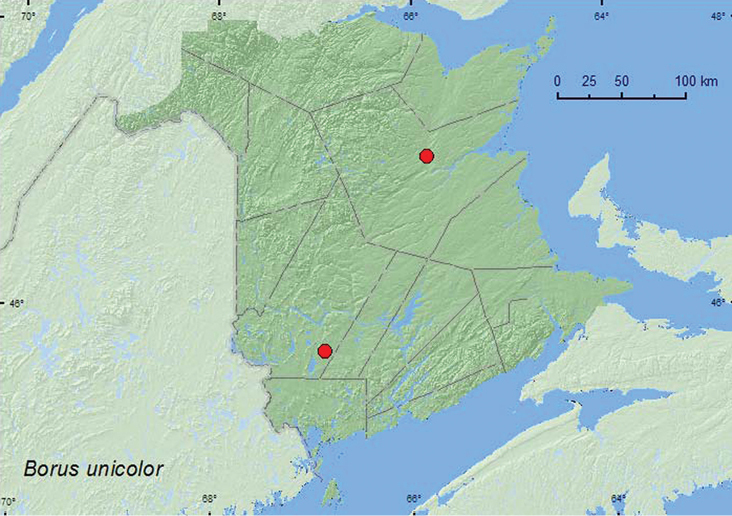
Collection localities in New Brunswick, Canada of *Boros unicolor*.

### Family Pythidae Solier, 1834

The Pythidae (the dead log beetles) of North America was reviewed by Pollock (1991, 2002c). Larvae of the Pythidae live in the subcortical region of dead coniferous trees (*Pytho*) or in the sapwood of conifer logs in the red rot stage (*Priognathus*) (Pollock 1991; Young 1991d). The larvae of *Pytho* are apparently xylophagus, as they have been reared solely on cambium of conifers (Pollock 1991). Adults may be predaceous based on characters of the mandibles, otherwise the food requirements of adults in this family are poorly known. Campbell (1991g) reported three species of Pythidae from New Brunswick; *Priognathus monilicornis* (Randall), *Pytho americanus* Kirby, and *Pytho strictus* LeConte. Majka (2006) added *Pytho niger* Kirby based on a specimen collected by W. McIntosh in Saint John during June 1900. Here, we report *Pytho seidlitzi* Blair for the first time for New Brunswick and the first recent records of *Pytho niger*.

#### Pytho niger

Kirby, 1837

http://species-id.net/wiki/Pytho_niger

Map 13

##### Material examined. 

**Additional New Brunswick records. Northumberland Co.**, 12 km SSE of Upper Napan near Goodfellow Brook, 46.8943°N, 65.3810°W, 23.V.2007, R. P. Webster, recent clear-cut**,** under bark of spruce log (6, NBM, RWC). **Sunbury Co.**, Acadia Research Forest, 45.9866°N, 66.3841°W, 19–25.V.2009, R. Webster & M.-A. Giguère, mature (100 year-old) red spruce forest with scattered red maple and balsam fir, Lindgren funnel trap (1, AFC). **York Co.**, Charters Settlement, 45.8331°N, 66.7410°W, 2.VI.2007, R. P. Webster, mature red spruce forest under bark of spruce log (on underside of log) (9, NBM, RWC); 15 km W of Tracy off Rt. 645, 45.6848°N, 66.8821°W, 19–25.V.2009, 1–8.VI.2009, 8–15.VI.2009, 15–21.VI.2009, R. Webster & M.-A. Giguère, old red pine forest, Lindgren funnel traps (5, AFC); same locality and habitat data but 10–16.V.2010, 16.V–4.VI.2010, R. Webster & C. MacKay, Lindgren funnel traps (4, AFC); 14 km WSW of Tracy, S of Rt. 645, 45.6741°N, 66.8661°W, 25.IV–10.V.2009, 10–26.V.2010, R. Webster & C. MacKay, old mixed forest with red and white spruce, red and white pine, balsam fir, eastern white cedar, red maple, and *Populus* sp., Lindgren funnel traps (2, AFC).

##### Collection and habitat data.

In New Brunswick, *Pytho niger* was collected in a mature red spruce, an old red pine, and old mixed forests. Adults with specific habitat data were collected from under bark of leaning, dead, red spruce tree trunks. Adults occurred on the underside of the logs. Adults were also captured in Lindgren funnel traps with some frequency. Pollock (1991) reported this species from white pine (*Pinus strobus* L.), jack pine, black spruce, and balsam fir. Adults were collected during April, May, and June in New Brunswick.

##### Distribution in Canada and Alaska.

AK, YK, NT, BC, AB, SK, MB, ON, QC, NB, NS, PE, NF (Campbell 1991g; Majka 2006).

**Map 13.**
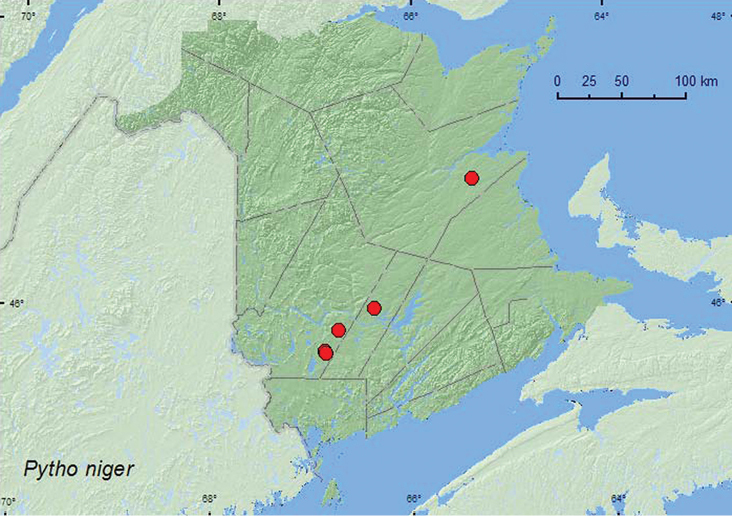
Collection localities in New Brunswick, Canada of *Pytho niger*.

#### Pytho seidlitzi

Blair 1925

http://species-id.net/wiki/Pytho_seidlitzi

Map 14

##### Material examined.

**New Brunswick, Sunbury Co.**, Acadia Research Forest, 45.9866°N, 66.3841°W, 28.IV–8.V.2009, R. Webster & M.-A. Giguère, mature (110-year-old) red spruce forest with scattered red maple and balsam fir, Lindgren funnel trap (1, RWC); same locality, forest type, and collectors, 13.V.2009, under bark of leaning dead red spruce, on underside of (leaning) trunk (1, RWC). **Restigouche, Co.**, Dionne Brook P.N.A, 47.9064°N, 68.3441°W, 31.V–15.VI.2011, M. Roy & V. Webster, old-growth white spruce and balsam fir forest (1, RWC). **York Co.**, Fredericton, 28.V.1929, L. J. Simpson (1, AFC); Charters Settlement, 45.8339°N, 66.7450°W, 15.V.2004, R. P. Webster, mixed forest under bark of spruce log (1, RWC); 15 km W of Tracy off Rt. 645, 45.6848°N, 66.8821°W, 26.IV–10.V.2010, R. Webster & C. MacKay, old red pine forest, Lindgren funnel trap (1, RWC); 14 km WSW of Tracy, S of Rt. 645, 45.6741°N, 66.8661°W, 26.IV–10.V.2009, R. Webster & C. MacKay, old mixed forest with red and white spruce, red and white pine, balsam fir, eastern white cedar, red maple, and *Populus* sp., Lindgren funnel trap (1, RWC).

##### Collection and habitat data.

In New Brunswick, this species was collected in a 110-year-old red spruce stand, an old (180-year-old) red pine forest, an old-growth white spruce and balsam fir forest (boreal forest), and in old mixed forests. Adults with habitat data recorded were collected from under bark of leaning, dead, red spruce trunks on the underside of the logs. A few adults were also captured in Lindgren funnel traps. Larval hosts include a variety of conifer species (Pollock 1991). Most adults were collected between late April and mid May, and one during late May and June.

##### Distribution in Canada and Alaska.

NT, BC, AB, MB, ON, QC, **NB**, NS (Campbell 1991g). This species was previously known from Cape Breton Island, Nova Scotia in the Maritime provinces (Campbell 1991g; Majka 2006). The above records from New Brunswick indicate a broader distribution for this species in the region.

**Map 14.**
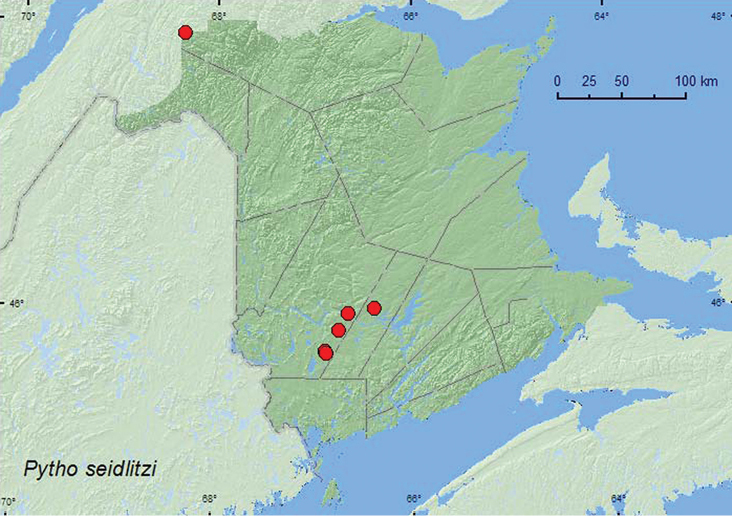
Collection localities in New Brunswick, Canada of *Pytho seidlitzi*.

### Family Pyrochroidae Latreille, 1806

The Pyrochroidae (the fire-colored beetles) of North America were reviewed by Young (2002b). Larval habitat associations of members of this family were described by Young (1991c, 2002b), and these references should be consulted for details with respect to the biology of species in this family. In general, most species are associated with moist, decomposing, subcortical conditions of dead coniferous and deciduous trees. Larvae of a few *Pedilus* species have been found within decaying vegetative material on or in soil (Young 2002b). Four species of Pyrochroidae were reported for New Brunswick by Bousquet (1991c) and Campbell (1991f). No additional species of this family were reported by Majka (2006) in his review of the fauna of the Maritime provinces. Here, we report three additional species from New Brunswick; *Neopyrochroa femoralis* (LeConte), *Pedilus canaliculatus* (LeConte), and *Pedilus elegans* (Hentz) (Table 1). The latter two species are newly recorded for the Maritime provinces.

### Subfamily Pedilinae Lacordaire, 1859

#### Pedilus canaliculatus

(LeConte, 1866)**

http://species-id.net/wiki/Pedilus_canaliculatus

Map 15

##### Material examined.

**New Brunswick, Carleton Co.**, Meduxnekeag Valley Nature Preserve, 46.1931°N, 67.6825°W, 8.VI.2005, R. P. Webster, floodplain forest, sweeping(1, RWC). **Restigouche Co.**, Stillwater Rd. at Stillwater Brook, 47.7320°N, 67.3376°W, 12.VI.2006, R.P. Webster, black spruce forest, on choke cherry flowers (9, RWC).

##### Collection and habitat data. 

Adults were common on choke cherry flowers along a roadside adjacent to a black spruce forest. One individual was swept from foliage in a floodplain forest. Adults were captured during June.

##### Distribution in Canada and Alaska.

QC, **NB** (Bousquet 1991c). Majka (2006) indicated that this species could be found in western or northern New Brunswick, as it occurred nearby in Maine.

**Map 15.**
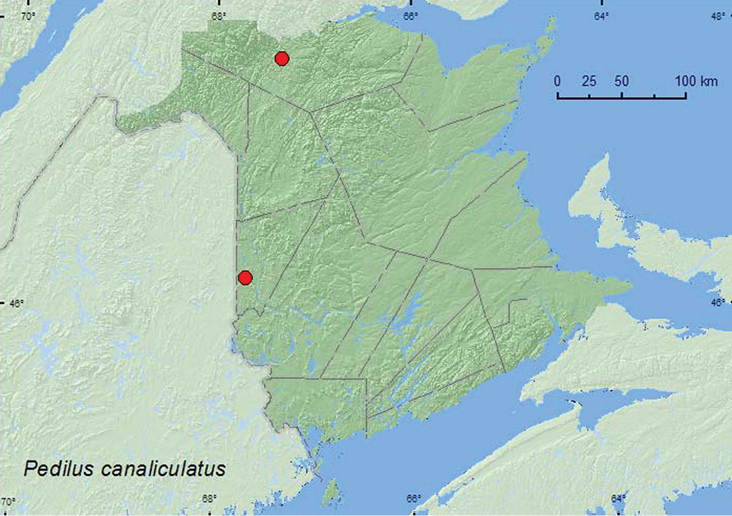
Collection localities in New Brunswick, Canada of *Pedilus canaliculatus*.

#### Pedilus elegans

(Hentz, 1830)**

http://species-id.net/wiki/Pedilus_elegans

Map 16

##### Material examined.

**New Brunswick, Carleton Co.**, Meduxnekeag Valley Nature Preserve, 46.1931°N, 67.6825°W, 7.VI.2007, R. P. Webster, floodplain forest, beating foliage of *Prunus virginiana* (1, RWC). **York Co.**, Canterbury, 45.8841°N, 67.6428°W, 8.VI.2004, D. Sabine & R. Webster, hardwood forest, sweeping foliage along woodland trail (3, RWC); Mazerolle Settlement, 45.8765°N, 66.8260°W, 8.VI.2008, R. P. Webster, beaver meadow, sweeping vegetation along brook margin (8, NBM, RWC); 15 km W of Tracy off Rt. 645, 45.6837°N, 66.8809°W, 10.VI.2009, R. P. Webster, old red pine forest, sweeping foliage (1, RWC).

##### Collection and habitat data.

This species was taken by beating foliage of choke cherry in a floodplain forest, sweeping foliage along a trail through a hardwood forest with sugar maple and American beech, and sweeping vegetation along a brook in a beaver meadow. Adults were collected during June.

##### Distribution in Canada and Alaska.

MB, ON, QC, **NB** (Bousquet 1991c).

**Map 16.**
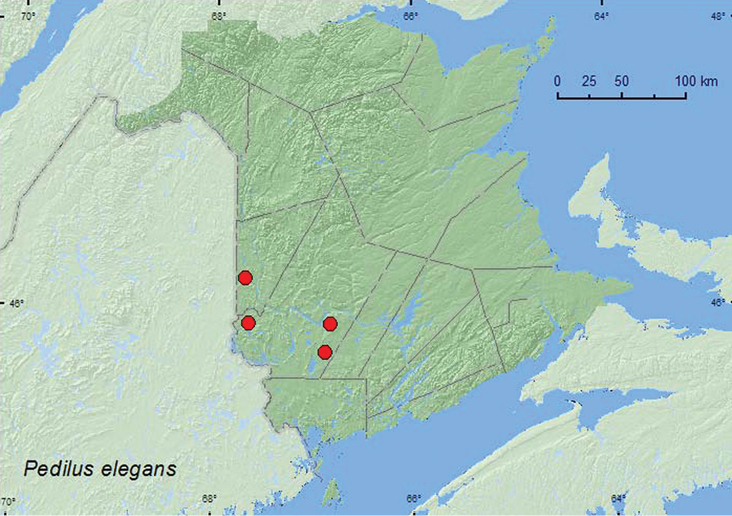
Collection localities in New Brunswick, Canada of *Pedilus elegans*.

### Subfamily Pyrochroinae Latreille, 1806

#### Neopyrochroa femoralis

(LeConte, 1855)

http://species-id.net/wiki/Neopyrochroa_femoralis

Map 17

##### Material examined.

**New Brunswick, Queens Co.**, Grand Lake near Scotchtown, 45.8762°N, 66.1816°W, 9.VII.2006, R. P. Webster, oak and maple forest, m.v. light (1, RWC); Grand Lake Meadows P.N.A., 45.8227°N, 66.1209°W, 15–29.VI.2010, 29.VI–12.VII.2010, R. Webster & C. MacKay, old silver maple forest with green ash and seasonally flooded marsh, Lindgren funnel traps (8, AFC, RWC); same locality data and forest type, 5–19.VII.2011, 19.VII–5.VIII.2011, M. Roy & V. Webster, Lindgren funnel traps in forest canopy (9, AFC, NBM).

##### Collection and habitat data.

One adult was collected at a mercury-vapor light in a red oak and maple forest near a lake; others were captured in Lindgren funnel traps deployed in an old silver maple swamp, including traps that were deployed in the forest canopy. Adults were collected during June, July, and August. Larvae occur under bark and decomposing wood of standing, dead, hardwood trees, usually near riparian areas (Young 2002b).

##### Distribution in Canada and Alaska.

ON, QC, **NB**, NS (Campbell 1991a; Majka 2006). Majka (2006) reported this species for the first time from the Maritime provinces, based on a specimen from Nova Scotia collected near Lake Kejimkujik in the Kejimkujik National Park. The above records indicate a broader distribution in the region.

**Map 17.**
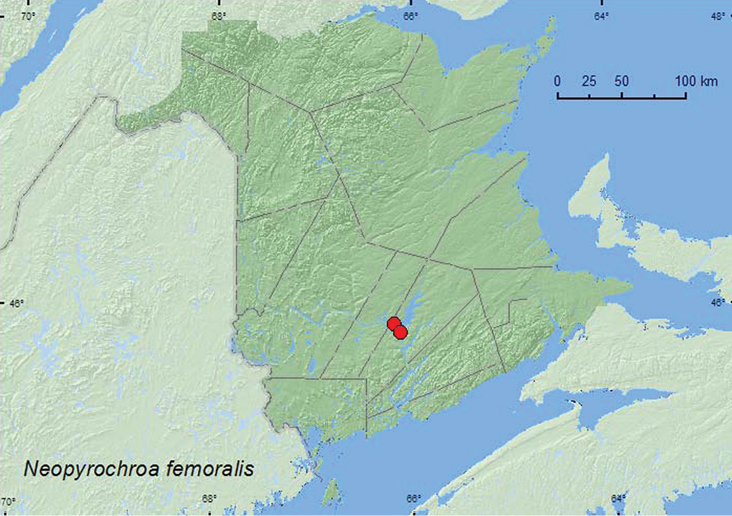
Collection localities in New Brunswick, Canada of *Neopyrochroa femoralis*.

### Family Anthicidae Latreille, 1819

The Anthicidae (the ant-like flower beetles) of North America was reviewed by Chandler (2002a). Members of this family are scavengers and predators on small arthropods. Many species are ground dwelling and typically occur on or under debris on exposed sand or soil or on vegetation (Chandler 2002a). Nine species of Anthicidae were reported from New Brunswick by Bousquet (1991b). *Sapintus pusillus* (LaFerté-Sénectère) was newly recorded from New Brunswick by Majka and Ogden (2006). Later, Majka (2011b) reviewed the Anthicidae of Atlantic Canada and reported *Amblyderus cervinus* LaFerté-Sénectère and *Amblyderus granularis* (LeConte) as new to the province. Here, we report five additional species from New Brunswick and remove one species from the faunal list (Table 1).

### Subfamily Eurygeniinae LeConte, 1862

#### Stereopalpus rufipes

Casey, 1895**

http://species-id.net/wiki/Stereopalpus_rufipes

Map 18

##### Material examined.

**New Brunswick, Queens Co.**, Grand Lake near Flowers Cove, 46.0196°N, 66.0246°W, 1.VII.2004, D. Sabine & R. Webster, lake shore, sweeping foliage (3, RWC); Grand Lake near Scotchtown, 45.8946°N, 66.1383°W, 28.VII.2005, R. Capozi & R. Webster, lake shore, on *Salix* sp. (1, RWC); same locality but 45.8762°N, 66.1816°W, 9.VII.2006, R. P. Webster, oak and maple forest, m.v. light (2, RWC).

##### Collection and habitat data.

This species wasswept from *Salix* sp. foliage and was captured at a mercury-vapor light deployed along a lake shore. Adults were collected during July.

##### Distribution in Canada and Alaska.

QC, **NB** (Bousquet 1991b).

**Map 18.**
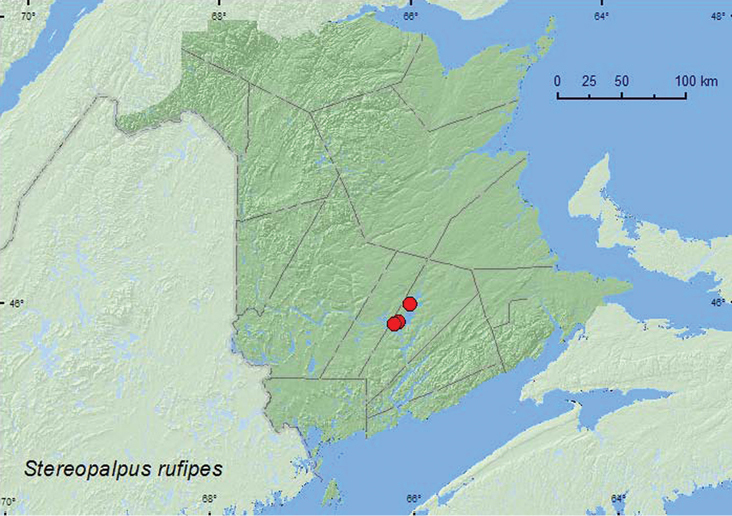
Collection localities in New Brunswick, Canada of *Stereopalpus rufipes*.

### Subfamily Anthicinae Latreille, 1819

#### Amblyderus granularis

(LeConte, 1850)

http://species-id.net/wiki/Amblyderus_granularis

##### Remarks.

*Amblyderus granularis* was reported from New Brunswick by Majka (2011b) on the basis of two specimens collected by R.P. Webster in Saint John (Saint John Co.) on 14 June 2002. These specimens were misidentified by C. G. Majka and are *Anthicus scabriceps* LeConte (determined by Donald Chandler). *Amblyderus granularis* is accordingly removed from the faunal list of New Brunswick.

#### Anthicus cervinus

LaFerté-Sénectère, 1849

http://species-id.net/wiki/Anthicus_cervinus

Map 19

##### Material examined.

**Additional New Brunswick records. York Co.**, Charters Settlement, 45.8395°N, 66.7391°W, 9.VII.2008, R. P. Webster, mixed forest, m.v. light (1, RWC).

##### Collection and habitat data.

In New Brunswick, *Anthicus cervinus* was collected at a mercury-vapor light in a mixed forest during July.

##### Distribution in Canada and Alaska.

NT, BC, AB, SK, MB, ON, QC, **NB** (Bousquet 1991b; Majka 2011b). Majka (2011b) reported this species from New Brunswick based on two specimens collected by W. McIntosh in Saint John (Saint John Co.) on 2 May 190X (early 1900s). The above record is the first recent record for this species from New Brunswick.

**Map 19.**
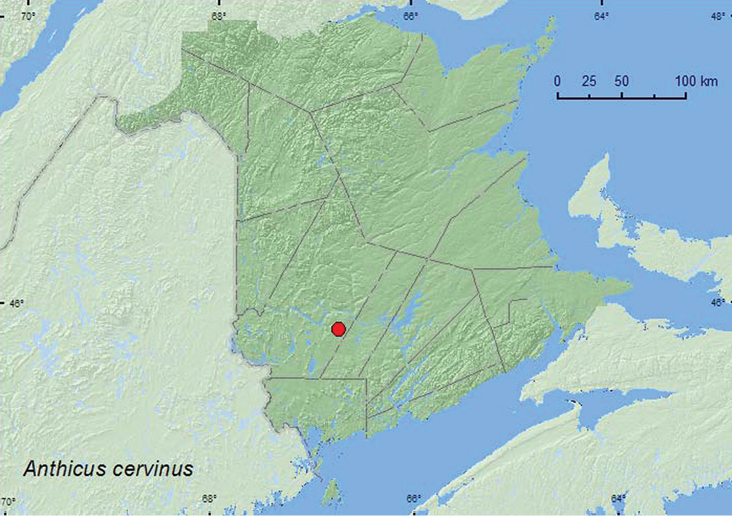
Collection localities in New Brunswick, Canada of *Anthicus cervinus*.

#### Anthicus haldemani

LeConte, 1852

http://species-id.net/wiki/Anthicus_haldemani

Map 20

##### Material examined.

**New Brunswick, Carleton Co.**, Jackson Falls, 46.2257°N, 67.7426°W, 14.V.2006, R. P. Webster, river margin, in drift material on ledge near falls (1, RWC); Jackson Falls, Bell Forest, 46.2150°N, 67.7201°W, 14.V.2006, R. P. Webster, river margin, in drift material near seepage area (3, NBM). **Queens Co.** Grand Lake at Stony Point, 46.0031°N, 66.0337°W, 17.VIII.2004, D. Sabine & R. Webster, lake shore on cobble beach, among cobbles (9, RWC).

##### Collection and habitat data.

In New Brunswick, *Anthicus haldemani* was collected from among cobblestones on a cobblestone lakeshore beach, in drift material on a ledge near a waterfall, and in drift material near a seepage area along a river margin. This species was collected from beach drift in Newfoundland (Majka 2011c). Adults were collected during May and August.

##### Distribution in Canada and Alaska.

NT, AB, SK, ON, QC, **NB**, NS, NF (Bousquet 1991b; Majka 2011b).

**Map 20.**
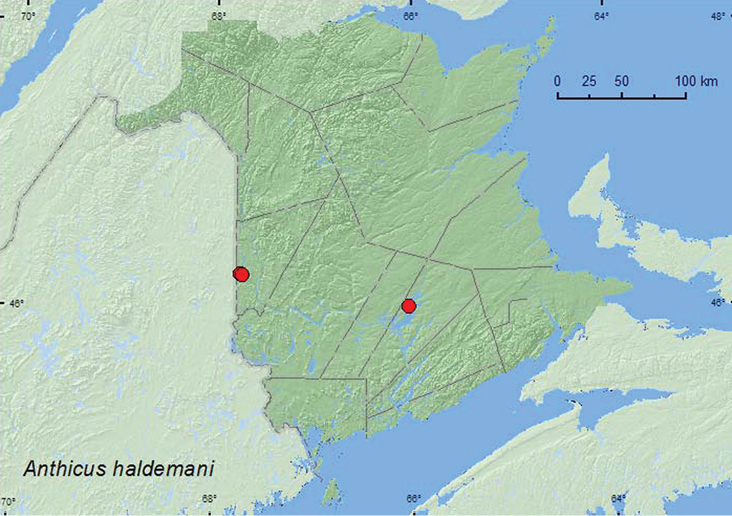
Collection localities in New Brunswick, Canada of *Anthicus haldemani*.

#### Anthicus melancholicus

LaFerté-Sénectère, 1848**

http://species-id.net/wiki/Anthicus_melancholicus

Map 21

##### Material examined.

**New Brunswick, Sunbury Co.** 9.5 km NE jct. 101 & 645, 45.7586°N, 66.6755°W, 30.VIII.2008, R. P. Webster, old field with open sandy areas, sweeping foliage (1, RWC).

##### Collection and habitat data.

This species was swept from foliage in an old field with open sandy areas. The adult was captured during late August.

##### Distribution in Canada and Alaska.

ON, QC, **NB** (Bousquet 1991b).

**Map 21.**
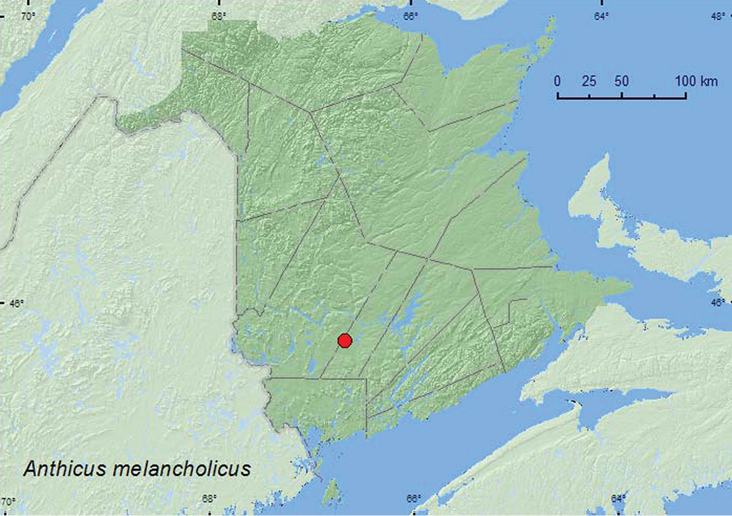
Collection localities in New Brunswick, Canada of *Anthicus melancholicus*.

#### Sapintus pubescens

(LaFerté-Sénectère, 1849)**

http://species-id.net/wiki/Sapintus_pubescens

Map 22

##### Material examined.

**New Brunswick, Queens Co.**, Grand Lake near Scotchtown, 45.8762°N, 66.1816°W, 3.VI.2007, R. P. Webster, oak and maple forest near lake shore, sweeping foliage (1, RWC). **Sunbury Co.** Maugerville, Portobello Creek N.W.A., 45.8992°N, 66.4248°W, 18.VI.2004, R. P. Webster, silver maple forest, u.v. light trap near slow (flowing) river (6, RWC). **York Co.**, Charters Settlement, 45.8395°N, 66.7391°W, 10.VI.2007, 1.VIII.2007, R. P. Webster, mixed forest, m.v. light (2, RWC).

##### Collection and habitat data

**.***Sapintus pubescens* was found in a red oak and red maple (*Acer rubrum* L.) forest near a lakeshore, in a silver maple forest, and in a mixed forest. Most individuals were captured in an ultraviolet light trap and at a mercury-vapor light. One individual was swept from foliage. Adults were collected during June and August.

##### Distribution in Canada and Alaska.

ON, QC, **NB** (Bousquet 1991b).

**Map 22.**
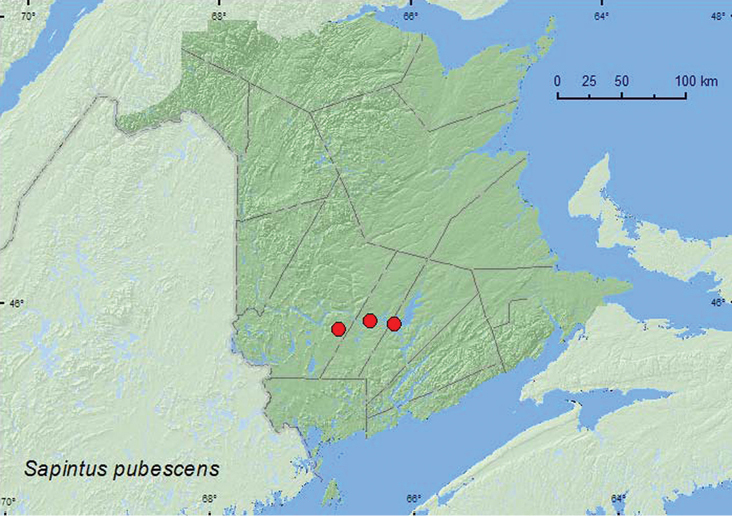
Collection localities in New Brunswick, Canada of *Sapintus pubescens*.

### Subfamily Notoxinae Stephens, 1829

#### Notoxus bifasciatus

(LeConte, 1852)**

http://species-id.net/wiki/Notoxus_bifasciatus

Map 23

##### Material examined.

**New Brunswick, Carleton Co.**, Lower Becaguimec Island, 46.2815°N, 67.5074°W, 16.VII.2008, R. P. Webster, island in Saint John River, sweeping low vegetation on cobblestone beach (14, NBM, RWC).

##### Collection and habitat data.

This species was swept from low vegetation (mostly *Apocynum cannabinum* L.) on a cobblestone area on an island in a large river. Adults were collected during July.

##### Distribution in Canada and Alaska.

MB**, NB** (Bousquet 1991b).

**Map 23.**
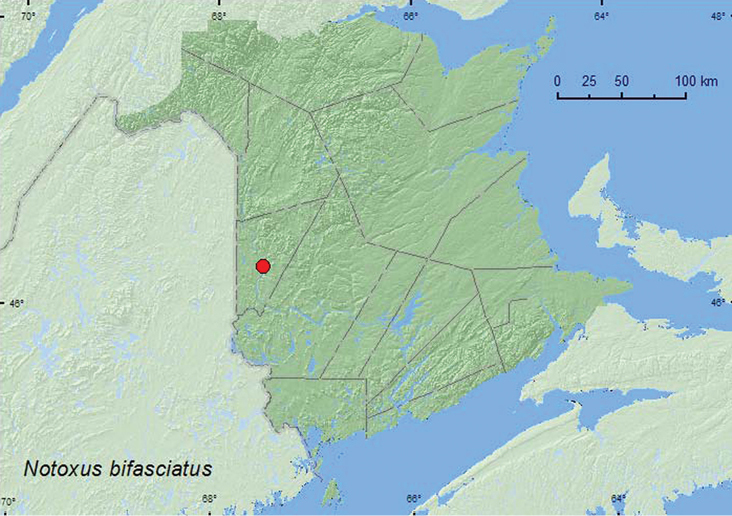
Collection localities in New Brunswick, Canada of *Notoxus bifasciatus*.

### Family Aderidae Csiki, 1909

The Aderidae (ant-like leaf beetles) of eastern North America was reviewed by Werner (1990) and in a general treatment of the North American members of the family by Chandler (2002b). Adults are usually found on the underside of leaves of shrubs and trees (Chandler 2002b). Larvae have been found in leaf litter and under bark (Young 1991b). Majka (2011b) reviewed the Aderidae of the Maritime provinces and reported two species new to the region. Only *Vanonus wickhami* Casey was reported from New Brunswick (Bousquet 1991a; Majka 2011b). Here, we report three additional species of Aderidae from New Brunswick, including *Vanonus huronicus* and *Zonantes fasciatus*, which are newly recorded for the Maritime provinces.

### Tribe Euglenesini Seidlitz, 1875

#### Zonantes fasciatus

(Melsheimer, 1846)**

http://species-id.net/wiki/Zonantes_fasciatus

Map 24

##### Material examined.

**New Brunswick, York Co.**, Charters Settlement, 45.8430°N, 66.7275°W, 20.VII.2008, R. P. Webster, regenerating mixed forest, sweeping foliage in brushy opening (1, RWC).

##### Collection and habitat data.

One individual was swept from foliage in a regenerating mixed forest in late July.

##### Distribution in Canada and Alaska.

ON, QC, **NB** (Werner 1990).

**Map 24.**
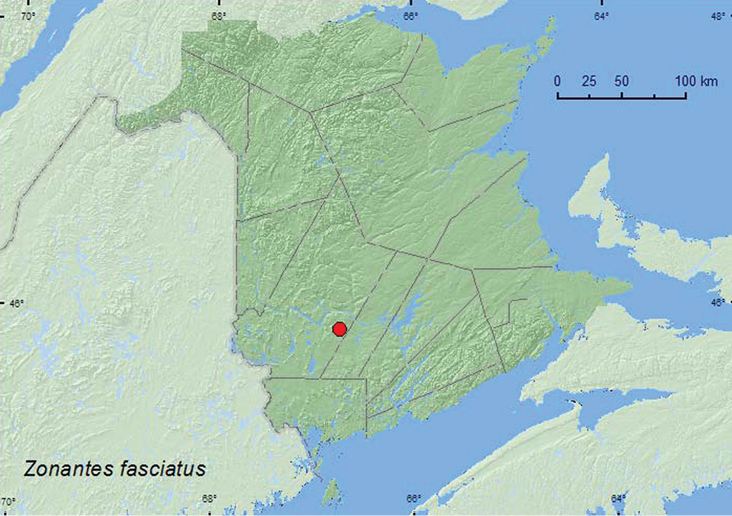
Collection localities in New Brunswick, Canada of *Zonantes fasciatus*.

#### Zonantes pallidus

Werner, 1990

http://species-id.net/wiki/Zonantes_pallidus

Map 25

##### Material examined.

**New Brunswick, Queens Co.**, Cranberry Lake P.N.A., 46.1125°N, 65.6075°W, 21–28.VII.2009, R. Webster & M.-A. Giguère, mature red oak forest, Lindgren funnel trap (1, RWC).

##### Collection and habitat data.

One individual was captured in a Lindgren funnel trap deployed in a red oak forest during July. Specimens from Nova Scotia were collected in forested localities with a car net (Majka 2011b).

##### Distribution in Canada and Alaska.

ON, QC, **NB**, NS (Werner 1990; Majka 2011b).

**Map 25.**
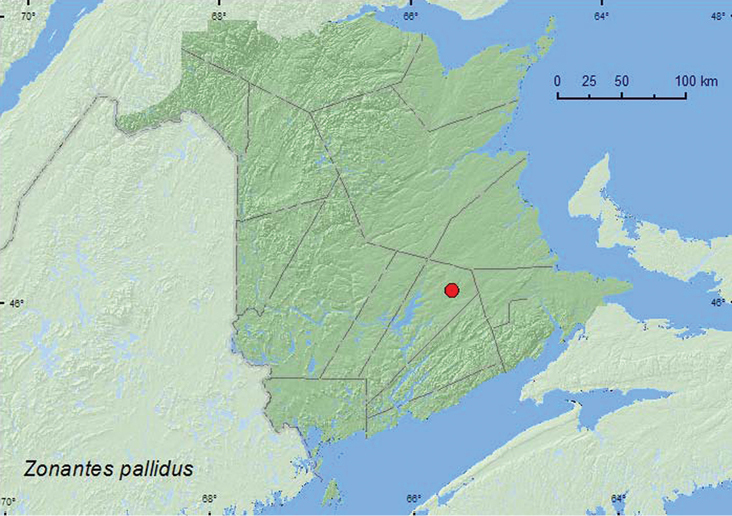
Collection localities in New Brunswick, Canada of *Zonantes pallidus*.

### Tribe Aderini Csiki, 1909

#### Vanonus huronicus

Casey, 1895**

http://species-id.net/wiki/Vanonus_huronicus

Map 26

##### Material examined.

**New Brunswick, Queens Co.**, Grand Lake Meadows P.N.A., 45.8227°N, 66.1209°W, 29.VI–12.VII.2010, R. Webster, C. MacKay, M. Laity, & R. Johns, old silver maple forest with green ask and seasonally flooded marsh, Lindgren funnel traps (3, CNC, RWC); Cranberry Lake P.N.A., 46.1125°N, 65.6075°W, 4–18.VIII.2011, M. Roy & V. Webster, mature red oak forest, Lindgren funnel trap (1, RWC).

##### Collection and habitat data.

Adults were captured in Lindgren funnel traps deployed in an old silver maple swamp and an old red oak forest. Adults in New Brunswick were collected during July and August.

##### Distribution in Canada and Alaska.

QC, **NB** (Laplante et al. 1991)**.**

**Map 26.**
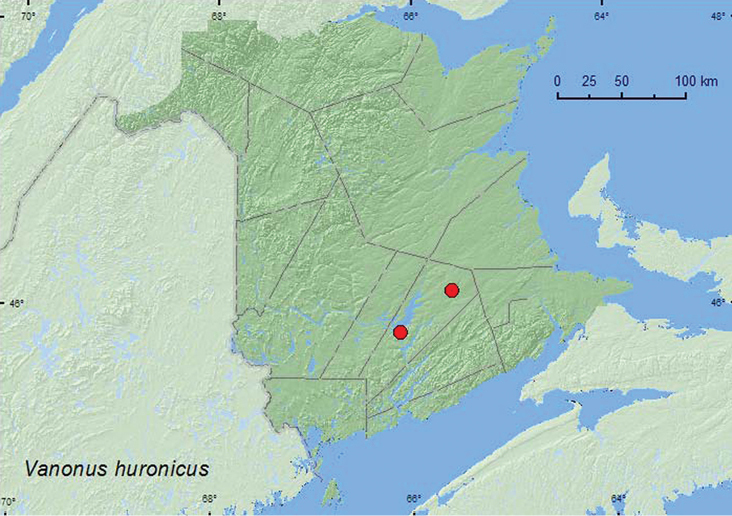
Collection localities in New Brunswick, Canada of *Vanonus huronicus*.

## Supplementary Material

XML Treatment for Cephaloon lepturides

XML Treatment for Cephaloon ungulare

XML Treatment for Nematoplus collaris

XML Treatment for Calopus angustus

XML Treatment for Asclera puncticollis

XML Treatment for Asclera ruficollis

XML Treatment for Ditylus caeruleus

XML Treatment for Epicauta pestifera

XML Treatment for Lytta sayi

XML Treatment for Meloe angusticollis

XML Treatment for Lacconotus punctatus

XML Treatment for Boros unicolor

XML Treatment for Pytho niger

XML Treatment for Pytho seidlitzi

XML Treatment for Pedilus canaliculatus

XML Treatment for Pedilus elegans

XML Treatment for Neopyrochroa femoralis

XML Treatment for Stereopalpus rufipes

XML Treatment for Amblyderus granularis

XML Treatment for Anthicus cervinus

XML Treatment for Anthicus haldemani

XML Treatment for Anthicus melancholicus

XML Treatment for Sapintus pubescens

XML Treatment for Notoxus bifasciatus

XML Treatment for Zonantes fasciatus

XML Treatment for Zonantes pallidus

XML Treatment for Vanonus huronicus
